# Rates of, and risk factors for, severe infections in patients with systemic autoimmune diseases receiving biological agents off-label

**DOI:** 10.1186/ar3397

**Published:** 2011-07-11

**Authors:** Cándido Díaz-Lagares, Roberto Pérez-Alvarez, Francisco J García-Hernández, María M Ayala-Gutiérrez, José Luis Callejas, Agustín Martínez-Berriotxoa, Javier Rascón, Luis Caminal-Montero, Albert Selva-O'Callaghan, Joaquim Oristrell, Carmen Hidalgo, Ricardo Gómez-de-la-Torre, Luis Sáez, Jesús Canora-Lebrato, María-Teresa Camps, Norberto Ortego-Centeno, María-Jesús Castillo-Palma, Manuel Ramos-Casals

**Affiliations:** 1 Laboratorio de Enfermedades Autoinmunes Josep Font, IDIBAPS, Hospital Clínic, C/Villarroel, Barcelona, 08036, Spain; 2 Servicio de Medicina Interna, Hospital Meixoeiro, Meixoeiro, Vigo, 36200, Spain; 3 Unidad de Colagenosis, Servicio de Medicina Interna, Hospital Virgen del Rocío, Avda. Manuel Siurot, Sevilla, 41013, Spain; 4 Unidad de Enfermedades Autoinmunes, Servicio de Medicina Interna, Hospital Carlos Haya, Avda. Carlos Haya, Málaga, 29010, Spain; 5 Unidad de Enfermedades Autoinmunes Sistémicas, Hospital San Cecilio, Avda. Dr. Olóriz, Granada, 18012, Spain; 6 Servicio de Medicina Interna, Hospital de Cruces, Plaza Cruces-Gurutzeta, Barakaldo, 48903, Spain; 7 Servicio de Medicina Interna, Hospital Son Dureta, C/Andrea Doria, Palma de Mallorca, 07014, Spain; 8 Servicio de Medicina Interna, Hospital Universitario Central de Asturias, C/Celestino Villamil, Oviedo, 33006, Spain; 9 Servicio de Medicina Interna, Hospital Vall d'Hebron, Passeig Vall d'Hebron, Barcelona, 08035, Spain; 10 Servicio de Medicina Interna, Hospital Parc Taulí, C/Parc Taulí, Sabadell, 08208, Spain; 11 Servicio de Medicina Interna, Hospital Virgen de las Nieves, Avda. Fuerzas Armadas, Granada, 18014, Spain; 12 Servicio de Medicina Interna, Hospital de Avilés, C/Camino Heros, Avilés, 33401, Spain; 13 Unidad de Enfermedades Autoinmunes, Servicio de Medicina Interna, Hospital Universitario Miguel Servet, Paseo Isabel la Católica, Zaragoza, 50009, Spain; 14 Hospital Universitario de Fuenlabrada, Camino del Molino, Fuenlabrada, 28942, Spain

**Keywords:** Infection rate, rituximab, infliximab, etanercept, adalimumab, systemic lupus erythematosus, Sjögren syndrome, vasculitis

## Abstract

**Introduction:**

The purpose of this observational study was to analyze the rates, characteristics and associated risk factors of severe infections in patients with systemic autoimmune diseases (SAD) who were treated off-label with biological agents in daily practice.

**Methods:**

The BIOGEAS registry is an ongoing Spanish prospective cohort study investigating the long-term safety and efficacy of the off-label use of biological agents in adult patients with severe, refractory SAD. Severe infections were defined according to previous studies as those that required intravenous treatment or that led to hospitalization or death. Patients contributed person-years of follow-up for the period in which they were treated with biological agents.

**Results:**

A total of 344 patients with SAD treated with biological agents off-label were included in the Registry until July 2010. The first biological therapies included rituximab in 264 (77%) patients, infliximab in 37 (11%), etanercept in 21 (6%), adalimumab in 19 (5%), and 'other' agents in 3 (1%). Forty-five severe infections occurred in 37 patients after a mean follow-up of 26.76 months. These infections resulted in four deaths. The crude rate of severe infections was 90.9 events/1000 person-years (112.5 for rituximab, 76.9 for infliximab, 66.9 for adalimumab and 30.5 for etanercept respectively). In patients treated with more than two courses of rituximab, the crude rate of severe infection was 226.4 events/1000 person-years. A pathogen was identified in 24 (53%) severe infections. The most common sites of severe infection were the lower respiratory tract (39%), bacteremia/sepsis (20%) and the urinary tract (16%). There were no significant differences relating to gender, SAD, agent, other previous therapies, number of previous immunosuppressive agents received or other therapies administered concomitantly. Cox regression analysis showed that age (*P *= 0.015) was independently associated with an increased risk of severe infection. Survival curves showed a lower survival rate in patients with severe infections (log-rank and Breslow tests < 0.001).

**Conclusions:**

The rates of severe infections in SAD patients with severe, refractory disease treated depended on the biological agent used, with the highest rates being observed for rituximab and the lowest for etanercept. The rate of infection was especially high in patients receiving three or more courses of rituximab. In patients with severe infections, survival was significantly reduced. Older age was the only significant predictive factor of severe infection.

## Introduction

In recent decades, the standard of care for patients with systemic autoimmune diseases (SAD) has been based on corticosteroids and immunosuppressive agents, although scientific evidence of their efficacy and safety relies principally on data from uncontrolled studies. The complexity of therapy in patients with SAD is increased by the large number of patients who do not respond to first-line therapies and those who relapse after initial clinical remission. In these patients, there is even less scientific evidence available for the use of second-line drugs, which are often prescribed according to individual clinical decisions. The emergence of biological therapies has increased the therapeutic armamentarium available in these complex situations. These therapies are used for a rapidly increasing number of cases of SAD [1] even though they are not yet licensed for this use by the US Food and Drug Administration (FDA) and the European Medicines Agency (EMEA). This off-label use is mainly employed to treat patients with either life-threatening situations, or severe involvement refractory or intolerant to standard therapy.

The risk of severe infection is a key factor in assessing the risks and benefits of using biologic agents to treat patients with SAD, especially as the use is off-label. Available data on the safety of biologic agents in SAD come from some randomized controlled trials (RCTs) and, especially, from a large number of small observational studies and case reports [2]. In RCTs, the prevalence of severe infection is less than 10% [3-16], but it is unclear whether the safety data obtained in these trials can be extrapolated to clinical practice. In RCTs, patients with associated processes or comorbidities are often excluded, meaning that the patient population tested is clearly different from patients treated in real-life conditions, in which biologic therapy is often more prolonged and patients have usually received long prior courses of corticosteroids and immunosuppressive agents. There is little information on the rate of severe infections in large series of patients with SAD treated off-label with biologic agents in clinical practice [17,18].

The purpose of this observational study was to analyze the rates, characteristics, and associated risk factors of severe infections in patients with SAD treated off-label with biologic agents in daily practice.

## Materials and methods

### Registry

The BIOGEAS (Spanish Study Group of Biological Agents in Autoimmune Diseases) registry is an ongoing Spanish prospective cohort study which since 2002 has been investigating the long-term safety and efficacy of the off-label use of biologic agents in adult patients with severe, refractory SAD [17]. By July 2010, the database included 344 patients reported by 21 Spanish departments of internal medicine. The SAD includes systemic lupus erythematosus (SLE), Sjögren syndrome, systemic sclerosis, polymyosistis, dermatomyositis, polyarteritis nodosa, Wegener granulomatosis, Churg-Strauss vasculitis, microscopic polyangiitis, giant cell arteritis, cryoglobulinemia, Behçet disease, sarcoidosis, adult-onset Still disease, antiphospholipid syndrome, relapsing polychondritis, idiopathic thrombocytopenic purpura, antisynthetase syndrome, and rheumatic polymyalgia. Diagnosis of SAD is based on the current international classification criteria for each disease. The inclusion criteria are: i) severe disease, defined as the development of potentially life-threatening clinical manifestations; or ii) refractory disease, defined as patients not achieving remission or relapsing, or patients with progressive disease despite optimal doses of corticosteroids and who have failed with at least two consecutive therapies considered as the standard of care for the corresponding autoimmune disease.

### Data collection

Data were collected at baseline (time of first administration of the drug), at six months and then every 12 months or at disease relapse, using an electronic case report form. To minimize possible interobserver bias, the inclusion criteria and variables of the protocol were agreed by all participating centers. The study was approved by the Ethics Committee of the coordinating center (Hospital Clinic, Barcelona, Spain) and then by each participating center. Written informed consent was obtained from all patients.

### Baseline assessment

Baseline information was collected on all patients in the BIOGEAS registry at the initiation of biologic therapy and included demographic data, disease duration, previous therapies, biologic agent, dosage, number of infusions/subcutaneous administrations, concomitant drugs, therapeutic response, relapses, retreatments and switches, adverse events, and outcomes.

### Definition of severe infection

Severe infections were defined according to previous studies [19,20] as those that required intravenous treatment or that led to hospitalization or death. Infections were classified by anatomic site and by microorganism.

### Statistical analysis

Patients contributed person-years of follow up for the period in which they were treated with biologic agents. Patients who later switched to other biologic agents, in which case infection was attributed to the last administered therapy, and patients who were re-treated, contributed separate events and person-years for each different drug or therapy cycle; person-years were calculated from the first day of administration of the biologic agent to the date of the last administered dose until July 2010, drug discontinuation, or death. In patients who discontinued biologic therapy, infection rates were calculated according to an exposure period-at-risk model used in recent studies in rheumatic diseases [19,20]. This type of model is in line with the recent European League Against Rheumatism (EULAR)recommendations on the reporting of safety data in biologic registers [21], in order to address the possible impact of an ongoing attributable risk beyond the drug discontinuation date [21]. This model consists of adding a lag window period which, in our study, was defined according to the biologic agent used. For rituximab, infections were included if they occurred during the 12 months after each infusion (initial treatment and/or each subsequent infusion) [18]. For the remaining agents, overwhelmingly anti-TNF agents, the exposure period was defined as the active treatment period plus a 90-day lag window [19].

Rates of serious infections are presented as events/1,000 person-years and 95% confidence intervals (CIs). Categorical data were compared using the chi squared and Fisher's exact tests. Continuous variables were analyzed with the Student's t-test in large samples of similar variance, with results indicated as mean * standard error of the mean (SEM), and with the nonparametric Mann-Whitney U-test for small samples, with results indicated as median and interquartiles. A two-tailed value of *P *< 0.05 was taken to indicate statistical significance. When independent variables appeared to have statistical significance in the univariate analysis, they were included in a multivariate Cox regression analysis using a backward stepwise method allowing adjustment for the variables that were statistically significant (*P *< 0.05) in the univariate analysis. The hazard ratios (HR) and their 95% CI obtained in the adjusted regression analysis were calculated. Kaplan-Meier survival curves of patients with and without severe infections were compared using the log-rank and Breslow tests. The statistical analysis was performed using the SPSS program (IBM SPSS Statistics 19.0).

## Results

### Baseline characteristics

The baseline characteristics are shown in Table 1. A total of 344 patients with SAD treated with biologic agents off-label were included in the registry until July 2010. There were 270 (78%) women and 74 (22%) men, with a mean age of 42.74 ± 0.83 (range 16 to 82) years. The main SAD were SLE in 140 (41%) cases, systemic vasculitis in 50 (14%), inflammatory myopathies in 38 (11%), Sjögren syndrome in 23 (7%), and Behçet disease in 31 (9%). Previous therapies included corticosteroids in 332 (97%) cases, cyclophosphamide in 172 (50%), methotrexate in 109 (32%), mycophenolate in 98 (28%), azathioprine in 97 (28%), and intravenous immunoglobulins in 89 (26%). Biologic therapies were administered due to life-threatening situations in 48 (14%) patients. The first biologic therapy included rituximab in 264 (77%) patients, infliximab in 37 (11%), etanercept in 21 (6%), adalimumab in 19 (5%), and other agents in 3 (1%). Concomitant therapies are summarized in Table 1. Sixty-one (18%) patients were treated with two courses of biologics, 14 (4%) with three courses and 8 (2%) with four courses. In 33 (10%) patients, the first biologic agent was switched to another agent.

**Table 1:** Baseline characteristics of 344 patients with systemic autoimmune diseases treated with biological agents

	*n *= 344
Females	270 (78%)

Mean age (years)	42.74 ± 0.83
Mean time of follow up (months)	26.76 ± 1.14 (0.5-105)
Autoimmune diseases	
- Systemic lupus erythematosus	140 (41%)
- Systemic vasculitis	50 (14%)
- Inflammatory myopathies	38 (11%)
- Behçet disease	31 (9%)
- Sjögren syndrome	23 (7%)
- Other diseases*	62 (18%)
Previous therapies (ever exposed)	
- Oral corticosteroids	332 (97%)
- Methylprednisolone pulses	86 (25%)
- Immunosuppressants	282 (82%)
- Intravenous immunoglobulins	89 (26%)
- Plasma exchange	10 (3%)
- Other therapies	109 (32%)
Cumulated dose/length of previous therapies	
- Mean length of prednisone therapy (years)	8.04 ± 0.65
- Cumulated dose of cyclophosphamide (gr)	7.18 ± 1.11
- Mean length of mycophenolate (years)	2.20 ± 0.39
- Mean length of azathioprine (years)	1.44 ± 0.35
- Number of previous immunosuppressants	
> = 3	73 (21%)
> = 4	25 (7%)
Biological agent	
- Rituximab	264 (77%)
- Infliximab	37 (11%)
- Etanercept	21 (6%)
- Adalimumab	19 (5%)
- Other	3 (1%)
Therapies administered concomitantly	
- Oral corticosteroids	312 (91%)
- Immunosuppressants	193 (56%)
- Intravenous immunoglobulins	14 (4%)
- Plasma exchange	11 (3%)
Time of exposure to biological (person-years)	
- Total time	495
- Exposure to rituximab	328
- Exposure to infliximab	52
- Exposure to etanercept	66
- Exposure to adalimumab	30
Retreatment	55 (16%)
Switch	33 (10%)

See the complete list of diseases included in the Methods section.

### Rates of severe infections

Forty-five severe infections, which required hospitalization and/or intravenous antibiotics and/or resulted in death, occurred in 37 patients after a mean follow up of 26.76 months (six patients had two or more severe infections). Infections occurred during the first six months after initiation of therapy (63%), between months 6 and 12 of therapy (13%), and after one year of initiation of therapy (24%). These infections resulted in four deaths. The crude rate of severe infections was 90.9 events/1,000 person-years (95% CI 66.31 to 121.64). The crude rates of serious infections according to treatment were 112.5 events/1,000 person-years for rituximab (95% CI 79.20 to 155.10), 76.9 events/1,000 person-years for infliximab (95% CI 20.96 to 196.95), 66.9 events/1,000 person-years for adalimumab (95% CI 8.10 to 241.55) and 30.5 events/1,000 person-years for etanercept (95% CI 3.69 to 110.02; Table 2). The crude rates of serious infections were 90.2 events/1,000 person-years for refractory patients (95% CI 25.80 to 365.30) and 94.8 events/1,000 person-years for patients presenting with life-threatening situations (95% CI 38.12 to 195.35).

**Table 2:** Rates of all severe infections, according to agent, number of courses, and main autoimmune diseases

	Patients	Person-years	Number of infections	Rate of infections/ 1,000 person-years	95% Confidence interval
TOTAL	344	495	45	90.90	66.31 to 121.64
RITUXIMAB					
- Total	264	328.83	37	112.52	79.20 to 155.10
- First course	211	252.17	30	119.00	88.03 to 169.80
- Second course	38	54.58	2	36.64	4.44 to 132.37
- Third or subsequent courses	15	22.08	5	226.40	73.50 to 528.50
ANTI-TNF AGENTS					
- Total	77	147.58	8	54.21	23.40 to 106.81
- Infliximab	37	52.00	4	76.92	20.96 to 196.95
- Adalimumab	19	29.91	2	66.85	8.10 to 241.55
- Etanercept	21	65.67	2	30.46	3.69 to 110.02
DISEASES					
- SLE	140	175.42	11	62.71	31.30 to 112.20
- Systemic vasculitis	50	74.75	11	147.16	73.50 to 263.30
- Inflammatory myopathies	38	60.42	7	115.86	46.60 to 238.70
- Behçet disease	31	61.25	2	32.65	3.95 to 117.95
- Sjögren syndrome	23	31.92	3	93.98	19.38 to 274.66
DISEASE/AGENT					
- Behçet disease/IFX	20	36.50	1	27.39	0.68 to 152.65
- Cryoglobulinemia/RTX	15	14.17	4	282.30	76.90 to 722.80
- Wegener granulomatosis/RTX	18	29.00	4	137.90	37.60 to 353.20
- SLE/RTX	139	171.08	11	64.30	32.09 to 115.04
- Inflammatory myopathies/RTX	27	32.17	2	62.18	7.53 to 224.61
- Sjögren syndrome/RTX	21	24.00	3	125.00	25.80 to 365.30
INDICATION					
- Refractory disease	296	421.17	38	90.22	63.85 to 123.84
- Life-threatening situation	48	73.83	7	94.81	38.12 to 195.35

IFX, infliximab; RTX, rituximab; SLE, systemic lupus erythematosus.

The crude rates of serious infections according to SAD were 147.2 events/1,000 person-years for systemic vasculitis (95% CI 73.50 to 263.30), 115.9 events/1,000 person-years for inflammatory myopathies (95% CI 46.60 to 238.70), 94.0 events/1,000 person-years for Sjögren syndrome (95% CI 19.38 to 274.66), 62.7 events/1,000 person-years for SLE (95% CI 31.30 to 112.20), and 32.6 events/1,000 person-years for Behçet disease (95% CI 3.95 to 117.95). Table 2 also includes the crude rates for the six most frequent disease/biologic agent combinations reported.

### Retreatment and switches

We analyzed the percentage and rates of severe infections in patients treated with more than one course of rituximab. The percentage of infection was 25 of 264 (9%) in the first course, 2 of 38 (5%) in the second course and 3 of 15 (20%) in the third and subsequent courses; the crude rates were 119.0, 36.6, and 226.4 events/1,000 person-years, respectively. Severe infections occurred in 2 of 33 (6%) patients in whom the biologic agent was switched.

### Pathogens

A pathogen was identified in 24 (53%) severe infections. Bacterial infections included *Streptococcus pneumoniae *(*n *= 3), *Escherichia coli *(*n *= 3), *Staphylococcus aureus *(*n *= 3), *Pseudomonas aeruginosa *(*n *= 2), *Staphylococcus sp *(*n *= 1), *Stenotrophomonas maltophilia *(*n *= 1), *Enterococcus faecalis *(*n *= 1), *Corynebacterium *(*n *= 1), and *Proteus mirabillis *(*n *= 1). Viral infections included cytomegalovirus (*n *= 3), and herpes simplex virus (*n *= 1). Fungal infections included candidiasis (*n *= 2), aspergillosis (*n *= 1), and *Pneumocystis jiroveci *infection (*n *= 1). There were three bacterial intracellular infections: *Mycobacterium tuberculosis *(*n *= 2) and *Listeria monocytogenes *(*n *= 1). Multibacterial infection was reported in one patient.

### Site of infection

The most common site of severe infection was the lower respiratory tract (39%), bacteriemia/sepsis (20%), the urinary tract (16%), the skin and soft tissues (9%), the gastrointestinal tract (5%), the central nervous system (5%), upper respiratory tract (5%), and the bones and joints (2%). Five (13%) patients presented separate infections in different sites.

### Risk factors for severe infections

Univariate analysis showed that patients who developed severe infections were older (49.14 vs 41.97 years, *P *= 0.008), had received methylprednisolone pulses less frequently (11% vs 27%, *P *= 0.043) and were treated concomitantly with cyclosporine A/tacrolimus more frequently (13% vs 5%, *P *= 0.041) in comparison with patients without severe infections (Table 3). There were no significant differences according to gender, SAD, agent, other previous therapies, number of previous immunosuppressive agents received, and other therapies administered concomitantly (Table 3). Multivariate Cox regression analysis showed that age (HR 1.026, 95% CI 1.005-1.047, *P *= 0.014) was significantly associated with an increased risk of severe infection.

**Table 3:** Main baseline characteristics of patients who developed severe infections in comparison with those who did not

	Severe infection *n *= 37	No severe infection *n *= 307	Bilateral *P *value
Females	32 (86%)	238 (77%)	0.289
Mean age (years)	49.14 ± 2.77	41.97 ± 0.86	0.008*
Autoimmune diseases			
- Systemic lupus erythematosus	11 (30%)	129 (42%)	
- Systemic vasculitis	8 (22%)	42 (14%)	
- Inflammatory myopathies	6 (16%)	32 (16%)	0.459
- Behçet disease	2 (5%)	29 (9%)	
- Primary Sjögren's syndrome	2 (5%)	21 (7%)	
- Other diseases	8 (22%)	54 (18%)	
Previous therapies (ever exposed)			
- Oral corticosteroids	35 (95%)	297 (97%)	0.377
- Methylprednisolone pulses	4 (11%)	82 (27%)	**0.043**
- Cyclophosphamide	23 (62%)	149 (48%)	0.163
- Mycophenolate	12 (32%)	86 (28%)	0.567
- Azathioprine	6 (16%)	91 (30%)	0.120
- Methotrexate	12 (32%)	97 (32%)	1.000
- Intravenous immunoglobulins	6 (16%)	83 (27%)	0.171
Cumulated dose/length of previous therapies			
- Mean length of prednisone therapy (years)	9.50 ± 2.89	7.93 ± 0.67	0.540
- Cumulated dose of cyclophosphamide (gr)	9.00	7.00 ± 1.20	0.629
- Mean length of mycophenolate (years)	0.96 ± 0.79	2.39 ± 0.44	0.236
- Mean length of azathioprine (years)	0.38 ± 0.28	1.56 ± 0.39	0.324
- Number of previous immunosuppressants			
> = 3	5 (13%)	68 (22%)	0.289
> = 4	2 (5%)	23 (7%)	1.000
Biological agent			
- Rituximab	29 (78%)	235 (76%)	
- Infliximab	4 (11%)	33 (11%)	
- Etanercept	2 (5%)	19 (6%)	0.982
- Adalimumab	2 (5%)	17 (5%)	
- Other	0 (0%)	3 (1%)	
Therapies administered concomitantly			
- Corticosteroids	35 (95%)	277 (90%)	0.554
- Cyclophosphamide	8 (22%)	95 (31%)	0.342
- Mycophenolate	5 (13%)	17 (5%)	0.073
- Azathioprine	0 (0%)	16 (5%)	0.235
- Methotrexate	5 (13%)	36 (12%)	0.788
- Cyclosporin A/Tacrolimus	5 (13%)	14 (5%)	**0.041**
- Intravenous immunoglobulins	2 (5%)	12 (4%)	0.653
Time of exposure to biological (person-years)			
- Total time	17.59 ± 2.16	17.23 ± 0.75	0.873
- Exposure to rituximab	15.13 ± 1.63	14.07 ± 0.47	0.471
- Exposure to infliximab	26.25 ± 15.4	15.27 ± 1.74	0.124
- Exposure to etanercept	12.50 ± 8.50	29.35 ± 3.84	0.246
- Exposure to adalimumab	26.00 ± 9.00	13.95 ± 2.52	0.183

*Statistically significant in the Cox regression multivariate model (hazard ratio 1.026, 95% confidence interval 1.005 to 1.047, *P *= 0.014).

### Mortality

**Figure 1:**
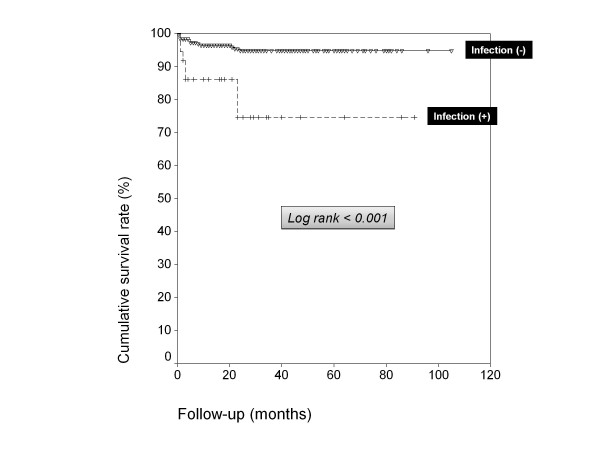
**Cumulative survival rate (%) in patients with systemic autoimmune diseases treated off-label with biologic agents (Kaplan-Meier plots) according to the development of infections**. Broken line + crosses = infected patients Continuous line + triangles = non-infected patients.

Figure 1 shows the survival curves of patients according to the development or not of severe infections (log-rank and Breslow tests < 0.001).

## Discussion

Biologic agents are currently used in patients with severe, refractory SAD even though they are not yet licensed for this use. Available scientific evidence comes from various RCTs but principally from a large number of observational studies and case reports. However, the results of uncontrolled studies and, especially, case reports may overstate the efficacy and understate the risks, due to the positive reporting and publication bias. One of the main concerns of the use of biologic agents is the potential risk of severe infections in patients with severe, refractory disease and a long-term history of the use corticosteroids and immunosuppressive agents, which *per se *increases the risk of infection. The best evidence on the safety of biological agents in SAD come from RCTs. In a pooled analysis of RCTs published to date (Table 4) [3-16], the global percentage of adverse events was significantly higher in patients receiving biologic agents in comparison with those receiving placebo (59.4% vs 48.7%, *P *< 0.001, HR 1.54, 95% CI 1.20-1.97). However, there were no significant differences either in the percentage of total number of infections (30.7% vs 29.9%, *P *= 0.79) or the percentage of severe infections (8.3% vs 7.6%, *P *= 0.69). However, the prevalence and rates of severe infections in SAD patients treated with biologic agents off-label in clinical practice has not yet been determined. For this reason, the Spanish Society of Internal Medicine set up a national multicenter prospective registry intended to determine the tolerance to and efficacy of biologic agents in clinical practice. The results of the present study show that 11% of patients developed severe infections, with a crude rate of 91 severe infections/1,000 person-years.

**Table 4:** Meta-analysis of adverse events in randomized controlled trials of biological agents in patients with systemic autoimmune diseases

	Disease	Patients (n)		Total side effects (n)		Total infections (n)		Severe infections (n)	
		*Placebo*	*Biologic*	*Placebo*	*Biologic*	*Placebo*	*Biologic*	*Placebo*	*Biologic*
Meijer et al [3]	Sjögren síndrome	10	20	4	16	4	11	NS	NS
Dass et al [4]	Sjögren síndrome	9	8	0	4	0	1	0	1
Merrill et al [5]	SLE	88	169	73	139	15	16	15	16
Stone et al [6]	ANCA-vasculitis	98	99	33	31	NS	NS	7	7
Jones et al [7]	ANCA-vasculitis	11	33	NS	NS	7	19	3	7
***RITUXIMAB***		216	329	110/205	190/296	26/118	47/230	25/206	31/309
				HR 1.55 (1.06-2.26)*		*HR 0.97 (0.80-1.17)*		*HR 0.81 (0.45-1.48)*	
Hoffman et al [8]	Giant cell arteritis	16	28	15	26	9	20	1	3
Salvarani et al [9]	Rheumatic polym.	28	23	5	7	0	1	0	1
Baughman et al [10]	Sarcoidosis	44	89	41	80	32	54	4	10
Mariette et al [11]	Sjögren síndrome	49	54	1	6	0	1	0	1
***INFLIXIMAB***		137	194	62 (45.2%	119 (61.3%)	41	76	5	15
				HR 1.92 (1.20-3.06)*		*HR 1.51 (0.92-2.47)*		*HR 2.21 (0.74-7.96)*	
Melikoglu et al [12]	Behcet	20	20	0	2	0	0	0	0
Baughman et al [13]	Sarcoidosis	9	9	5	2	2	2	0	0
Sankar et al [14]	Sjögren syndrome	14	14	1	2	1	0	0	0
WGET [15]	ANCA-vasculitis	91	89	51	51	45	44	NS	NS
Mtnez-Taboada et al [16]	Giant cell arteritis	9	8	7	8	4	4	0	0
***ETANERCEPT***		143	140	64 (44.7%)	65 (46.4%)	52 (36.4%)	50 (35.7%)	0 (0%)	0 (0%)
				*HR 1.07 (0.65-1.75)*		*HR 0.97 (0.58-1.63)*		*Not calculable*	
**TOTAL**		**496**	**663**	**236/485** **(48.7%)**	**374/630** **(59.4%)**	**119/398** **(29.9%)**	**173/564** **(30.7%)**	**30/395** **(7.6%)**	**46/554** **(8.3%)**
				HR 1.54 (1.20-1.97)*		*HR 1.04 (0.78-1.39)*		*HR 1.10 (0.67-1.84)*	

*P < 0.05; ANCA, anti-neutrophil antibodies; HR, hazard ratio; NS, not specified; SLE, systemic lupus erythematosus.

The highest rate of severe infections was observed in SAD patients treated with rituximab, which was used in nearly 80% of our patients, the majority of whom had SLE, systemic vasculitis, or Sjogren syndrome. Since its introduction, rituximab has been increasingly used in patients with SAD and there are more than 1,000 reported cases [22], overwhelmingly from uncontrolled studies and case reports. However, the percentage and rates of severe infections are unclear. The results of five reported RCTs in SAD patients treated with rituximab [3-7] showed a percentage of severe infections of 10%, very similar to that found in patients receiving placebo (12%) (Table 4) and the results of this study (11%). Recent data from the French AIR Registry [18] showed a higher rate in 136 SLE patients treated with rituximab (66 severe infections per 1,000 person-years), similar to the rate found in our SLE patients (63 per 1,000 person-years). Our study is the first to analyze rates of severe infection in patients with a wide variety of SAD. We found the highest rates for vasculitis and inflammatory myopathies (147 and 116 per 1,000 person-years, respectively) and the lowest rate for Behçet disease (30 per 1,000 person-years). In addition, we found that the rates of severe infection differed according to the number of rituximab courses received, with a significantly increased risk in patients receiving three or more courses of rituximab.

The rate of severe infections in SAD patients treated with anti-TNF agents was 54 per 1,000 person-years, and was much higher for the monoclonal antibodies (infliximab and adalimumab) than for the soluble receptor (etanercept), in line with some recent studies suggesting a lower rate of infections in patients treated with etanercept [23,24]. These rates are very similar to those reported in RCTs and postmarketing studies in patients with RA, which range between 17 and 64 per 1,000 person-years [25-30], although the differences between rheumatoid arthritis and the SAD studied here makes it difficult to compare infection rates. Infection of the lower respiratory or urinary tracts together with bacteremia/sepsis accounted for 75% of severe infections in our patients, with a microorganism being identified in more than 50% of episodes. Two patients treated with rituximab and no patients treated with anti-TNF agents developed tuberculosis. No patient developed progressive multifocal leukoencephalopathy.

One of the objectives of this study was to identify possible baseline risk factors for the development of severe infections in SAD patients treated with biologic agents. In patients with RA, identified risk factors for severe infection include age, extraarticular involvement, leukopenia, low baseline IgG levels, corticosteroid dosage, and comorbidities [31-34]. The largest prospective study [34] identified older age, rheumatoid arthritis-related extraarticular involvement, chronic lung disease and/or heart failure, and IgG levels below 6 g/L as independent variables associated with severe infections. Although the differences between this study and our results are significant (design, disease evaluated, drug licensed, and variables analyzed), it is noteworthy that both studies identified age as an independent variable associated with an increased risk of infection. However, we found no association between previous or concomitant use of corticosteroids and other immunosuppressive agents (cyclophosphamide, mycophenolate, methotrexate, or azathioprine) and an increased risk of severe infection.

Possible concerns in observational studies include selection bias (in our study, only refractory/severe patients were included). This patient profile makes it impossible to compare our results with those of controlled studies, a bias that, in our opinion, is very difficult to avoid due to the lack of licensing for biologic agents in autoimmune diseases. In addition, the lack of a control group of SAD patients treated with non-biologic therapies did not permit the risk of infection to be compared. However, in contrast to rheumatoid arthritis, it is very difficult to design a case-control study in this setting (SAD patients with life-threatening situations or refractory to standard therapies). Our study has other limitations, including the lack of evaluation of some baseline factors that could influence the risk of infection, such as laboratory parameters (neutrophil/lymphocyte counts, serum IgG levels), corticosteroid dosage, and baseline disease activity. However, our population study (patients with refractory disease or life-threatening situations) defines *per se *a group of patients with a high baseline disease activity and, in fact, re-analysis showed no significant differences between the crude rate of infection in these two groups. Nevertheless, in spite of these limitations, we believe that the recruitment of nearly 350 patients with SAD treated off-label with biologic agents is significant and permits useful information on the characteristics and rates of severe infection associated with the off-label use of biologic agents in patients with severe, refractory disease to be obtained.

## Conclusions

In conclusion, we found a crude rate of severe infection of 91 events per 1,000 person-years in a large series of patients with SAD treated off-label with biologic therapies, with the highest rate being observed for rituximab and the lowest for etanercept. The only predictive factor independently associated with severe infection was age. Although awaiting the licensing of biologic agents for SAD, an assessment of the risk of serious adverse events versus the benefits of treatment should be made on an individual basis.

## Abbreviations

SAD: systemic autoimmune diseases; FDA: Food and Drug Administration; EMEA: European Medicines Agency; RCT: randomized controlled trial; CI: confidence interval; SEM: standard error of the mean; HR: hazard ratio; SLE: systemic lupus erythematosus; TNF: tumor necrosis factor.

## Competing interests

The BIOGEAS Study group has received educational grants from Roche and Abbott supporting the design and maintenance of the webpage [35]. All authors have declared no conflicts of interest. None has received grants from these laboratories or conducted clinical trials with rituximab or etanercept as principal investigators or received honoraria as an Advisory Board member for Roche and Abbott. The financial support of Roche and Abbott is exclusively limited to maintaining the BIOGEAS webpage.

## Authors' contributions

CDL, RPA, and MRC initiated the study and contributed to design, statistical analysis, and drafting and revising the manuscript. All authors contributed to data processing, including data analysis. All authors read and approved the final manuscript.
